# Lysosomal Membrane Permeabilization is an Early Event in Sigma-2 Receptor Ligand Mediated Cell Death in Pancreatic Cancer

**DOI:** 10.1186/1756-9966-31-41

**Published:** 2012-05-02

**Authors:** John R Hornick, Suwanna Vangveravong, Dirk Spitzer, Carmen Abate, Francesco Berardi, Peter Goedegebuure, Robert H Mach, William G Hawkins

**Affiliations:** 1 Department of Surgery, Washington University School of Medicine, S. Euclid Avenue, St. Louis, MO, USA; 2 Alvin J. Siteman Cancer Center, Washington University School of Medicine, S. Euclid Avenue, St. Louis, MO, USA; 3 Division of Radiology, Washington University School of Medicine, St. Louis, MO, USA; 4 Dipartimento Farmacochimico, Università degli Studi di Bari Aldo Moro, di Bari Aldo Moro, Italy

**Keywords:** sigma-2 receptor, pancreatic cancer, caspase-3, lysosomal membrane permeablization

## Abstract

**Background:**

Sigma-2 receptor ligands have been studied for treatment of pancreatic cancer because they are preferentially internalized by proliferating cells and induce apoptosis. This mechanism of apoptosis is poorly understood, with varying reports of caspase-3 dependence. We evaluated multiple sigma-2 receptor ligands in this study, each shown to decrease tumor burden in preclinical models of human pancreatic cancer.

**Results:**

Fluorescently labeled sigma-2 receptor ligands of two classes (derivatives of SW43 and PB282) localize to cell membrane components in Bxpc3 and Aspc1 pancreatic cancer cells and accumulate in lysosomes. We found that interactions in the lysosome are critical for cell death following sigma-2 ligand treatment because selective inhibition of a protective lysosomal membrane glycoprotein, LAMP1, with shRNA greatly reduced the viability of cells following treatment. Sigma-2 ligands induced lysosomal membrane permeabilization (LMP) and protease translocation triggering downstream effectors of apoptosis. Subsequently, cellular oxidative stress was greatly increased following treatment with SW43, and the hydrophilic antioxidant N-acetylcysteine (NAC) gave greater protection against this than a lipophilic antioxidant, α-tocopherol (α-toco). Conversely, PB282-mediated cytotoxicity relied less on cellular oxidation, even though α-toco did provide protection from this ligand. In addition, we found that caspase-3 induction was not as significantly inhibited by cathepsin inhibitors as by antioxidants. Both NAC and α-toco protected against caspase-3 induction following PB282 treatment, while only NAC offered protection following SW43 treatment. The caspase-3 inhibitor DEVD-FMK offered significant protection from PB282, but not SW43.

**Conclusions:**

Sigma-2 ligand SW43 commits pancreatic cancer cells to death by a caspase-independent process involving LMP and oxidative stress which is protected from by NAC. PB282 however undergoes a caspase-dependent death following LMP protected by DEVD-FMK and α-toco, which is also known to stabilize the mitochondrial membrane during apoptotic stimuli. These differences in mechanism are likely dependent on the structural class of the compounds versus the inherent sigma-2 binding affinity. As resistance of pancreatic cancers to specific apoptotic stimuli from chemotherapy is better appreciated, and patient-tailored treatments become more available, ligands with high sigma-2 receptor affinity should be chosen based on sensitivities to apoptotic pathways.

## Introduction

Sigma receptors have been intensely studied for their applications in both neuropharmacology and oncology. Two subtypes of sigma receptors are known, sigma-1 and −2, which were classically characterized by differences in their relative binding affinity of ^3^ H]-(+)-pentazocine (sigma-1 > sigma-2) [1] and ^3^ H]-1,3 di-ortho-tolylguanidine (^3^ H]-DTG) (sigma-1 = sigma-2) [2] because of lack of genetic identification of the sigma-2 receptorfor many years. However, we have recently identified progesterone receptor membrane component 1 (PGRMC1) protein complex as containing the sigma-2 receptor binding site [3]and others recently found PGRMC1/sigma-2 to be elevated in tumors and serum of lung cancer patients [4].

**Table 1:** **Pancreatic cancer cell line viability, IC**_**50**_**(μM), following sigma-2 receptor ligand treatment (24 hr)**

	**Panc02**			**Bxpc3**			**Aspc1**		
	Mean	SEM	n	Mean	SEM	n	Mean	SEM	n
SV119	92	10	4	97	16	3	192	41	4
SW43	26	5	4	56	14	3	65	12	4
PB28	73	10	4	96	16	3	244	48	4
PB282	79	16	4	82	20	3	135	10	4

Sigma-2 receptors are overexpressed in multiple tumor types including breast, pancreas, neuroblastoma, bladder, and lung as reviewed [5], which has allowed further development of these ligands as radiotracers for the imaging of cancer [6]. In addition, various sigma-2 receptor ligands have been extensively studied for their effectiveness in the treatment of solid tumors due to their preferential uptake in proliferating cells [7]. We have previously shown that sigma-2 receptors are upregulated in pancreatic cancer, that sigma-2 ligands can induce caspase-3-mediated apoptisis, and are effective in preclinical models of pancreatic cancer [8-10].

Sigma-2 receptor ligands that have been investigated for efficacy in the treatment of cancer induce apoptosis in caspase-3 dependent and independent manners, but the exact mechanism of cell death is still not well characterized. For example, in SK-N-SH neuroblastoma cells caspase-3 was not activated by CB-64D [11], nor did caspase inhibitors afford protection against cell death in MCF-7 breast cancer cells [12]. Caspase-3 is however activated in MCF-7 [13] and in murine pancreatic adenocarcinoma Panc02cells [10] bysiramesine, though caspase-3 inhibitor did not rescue viability in either case. With another compound, PB28, no caspase-3 activity was observed in MCF-7 [14] or SK-N-SH cells [15].

Thus, while various sigma-2 receptor ligands are capable of inducing apoptosis in tumor cells, the activation of caspase-3 and upstream signaling events leading to this appear to be specific to particular ligand and cell type. In this study, we sought to more closely study the apoptotic pathway induced by a number of structurally distinct sigma-2 receptor ligands in pancreatic cancer, which have proven efficacious in preclincal models. With knowledge of chemotherapy resistance to apoptotic stimuli depending on different mechanisms, we may more appopriately choose effective therapies.

## Results

### Structurally distinct sigma-2 receptor ligands inhibit growth of pancreatic cancer

**Figure 1:**
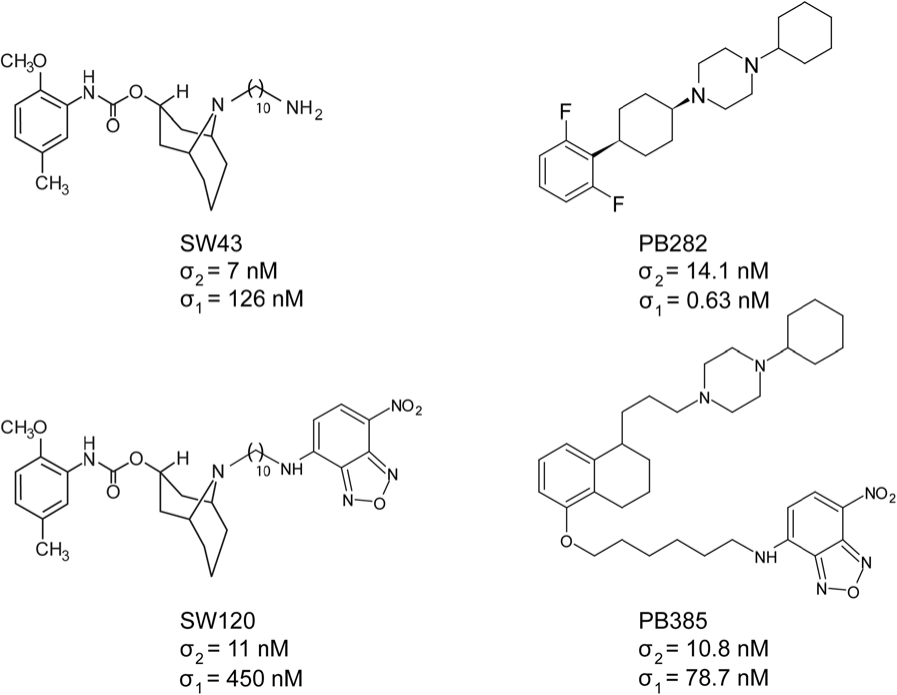
** Structures.** Sigma-2 receptor ligands SW43 and SW120, derivatives of N-(9-(6-aminohexyl)-9-azabicyclo[3.3.1]nonan-3α-yl)-N-(2-methoxy-5-methylphenyl) carbamate hydrochloride (SV119), and PB282 and PB385, derivatives of 1-cyclohexyl-4-[3-(5-methoxy-1,2,3,4-tetrahydro-naphthalen-1-yl)propyl]-piperazine dihydrochloride (PB28). Affinity to sigma-1/2 (σ2) receptor given by Ki (nM).

**Figure 2:**
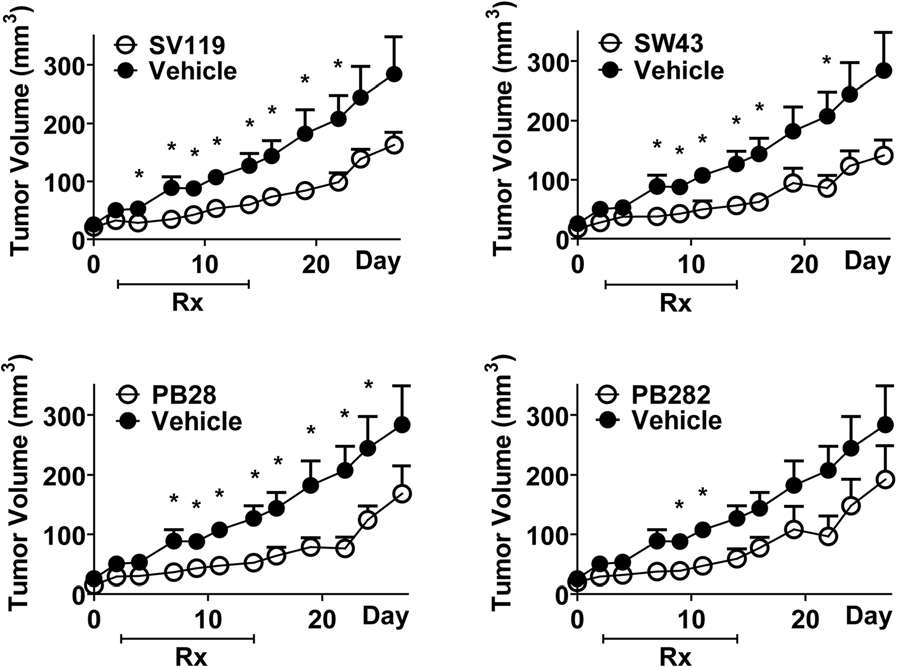
** In vivo efficacy of sigma-2 receptor ligands.** Athymic nude mice inoculated subcutaneously with 1x106 Bxpc3 cells were treated daily with sigma-2 receptor ligands SV119, SW43, PB28, or PB282 when tumors reached an average of 5 mm in diameter. Data represents mean ± SEM, n = 7–10 per group, * p < 0.05.

Multiple structurally distinct compounds (Figure 1) with high affinity for sigma-2 receptors were tested for cytotoxicity against multiple pancreatic cancer cell lines in vitro (Table1) and screened for efficacy in a mouse model of pancreatic cancer with Panc02 cells (Additional file 1: figure S1). Compounds were further tested in athymic nude mice bearing human Bxpc3 subcutaneous tumorsand treated daily with equimolar doses of these sigma-2 receptor ligands. These mice with established tumors were treated for eleven days and compared to vehicle, SV119, SW43, PB28, and PB282 each significantly decreased tumor volume (Figure 2).

### Distinct sigma-2 receptor ligands induce lysosomal membrane permeabilization (LMP)

**Figure 3:**
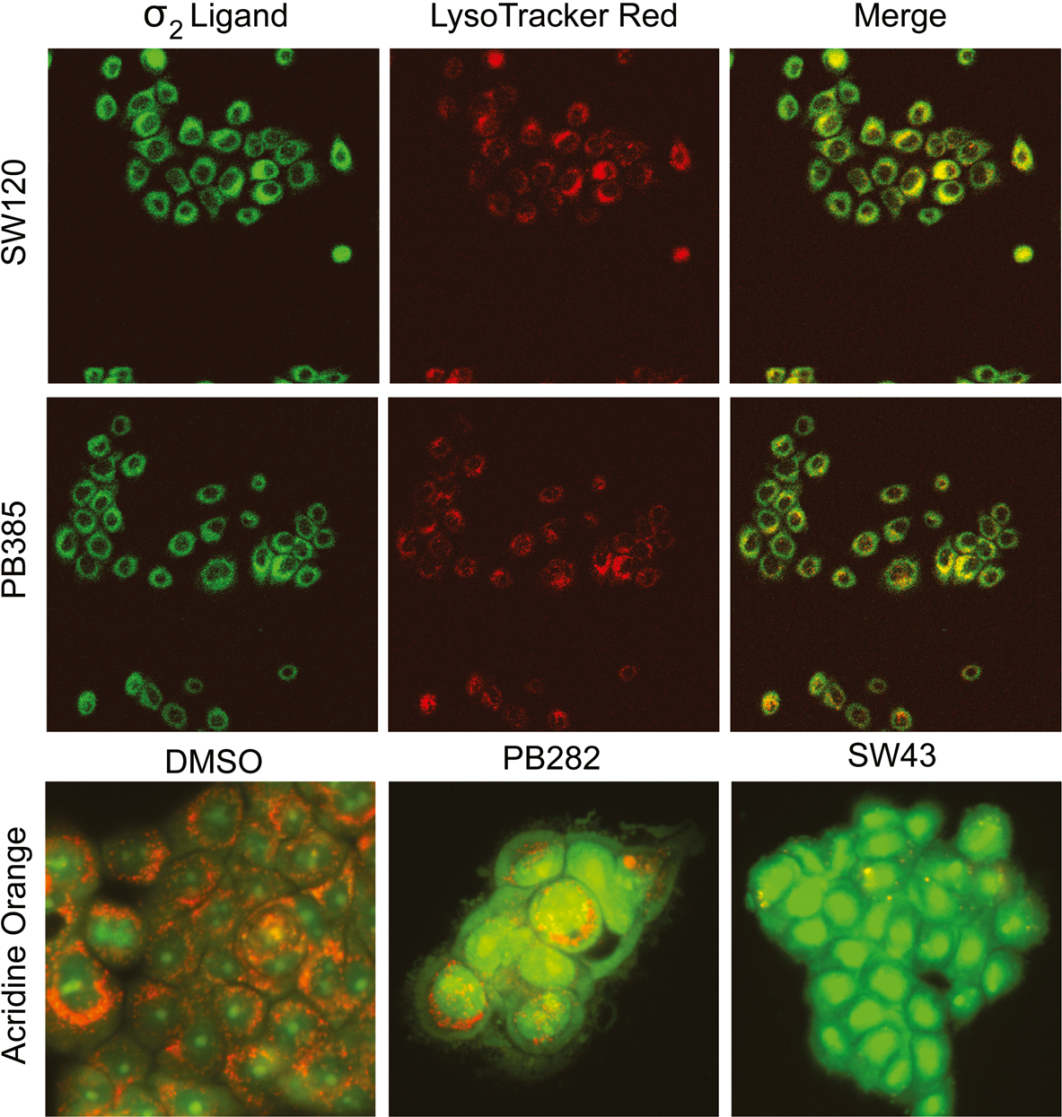
** Sigma-2 receptor ligands localize to lysosomes and induce lysosomal membrane permeabilization.** Upper two rows, confocal images of SW120 and PB385 (100 nM) in Bxpc3 cells, left (green), LysoTracker Red (25 nM), middle (red), and overlay right. Bottom row, acridine orange (2 μg/mL) staining for lysosomal integrity in Bxpc3 cells treated with vehicle, left, PB282 (30 μM) middle, or SW43 (30 μM) right for one hour. Scale bar = 20 μm.

Recently characterized fluorescent sigma-2 receptor ligands SW120 (derivative of SW43) [16] and PB385 (derivative of PB282) [17], colocalize with LysoTracker Red in Bxpc3 pancreatic cancer cells by confocal microscopy (Figure 3), and also appreciated by fluorescent microscopy in Bxpc3 and Aspc1 (Additional file 2 figure S2A). Bxpc3 cells preloaded with acridine orange (AO, 2 μg/mL), had decreased retention of orange dye in the lysosome (Figure 3, bottom panel) following treatment with SW43 and PB282, and displayed increased green fluorescence as dye escaped and bound with nucleic acids. Similar results were observed in Aspc1 cells (Additional file 2 figure S2A). The pH gradient of the lysosome is actively driven by a V-Type ATPase H^+^ pump [18], and its inhibition with concanamycin A (CMA) prevented dye retention in the lysosome. As well, hydroxychloroquine (HCQ), originally used for treatment of malaria [19] and extensively studied as a lysosomotropic detergent [20] showed decreased dye retention (positive control) (Additional file 3 figure S3A). SV119 and PB28, with high affinity to sigma-2 receptors, displayed leakeage of lysosomal dyes by acridine orange,and leakage by all compounds was confirmed with LysoTracker Green (Additional file 3 figure S3B).

### Compromising lysosomal membrane integrity sensitizes pancreatic cancer cells to sigma-2 receptor ligand mediated LMP and cell death

**Figure 4:**
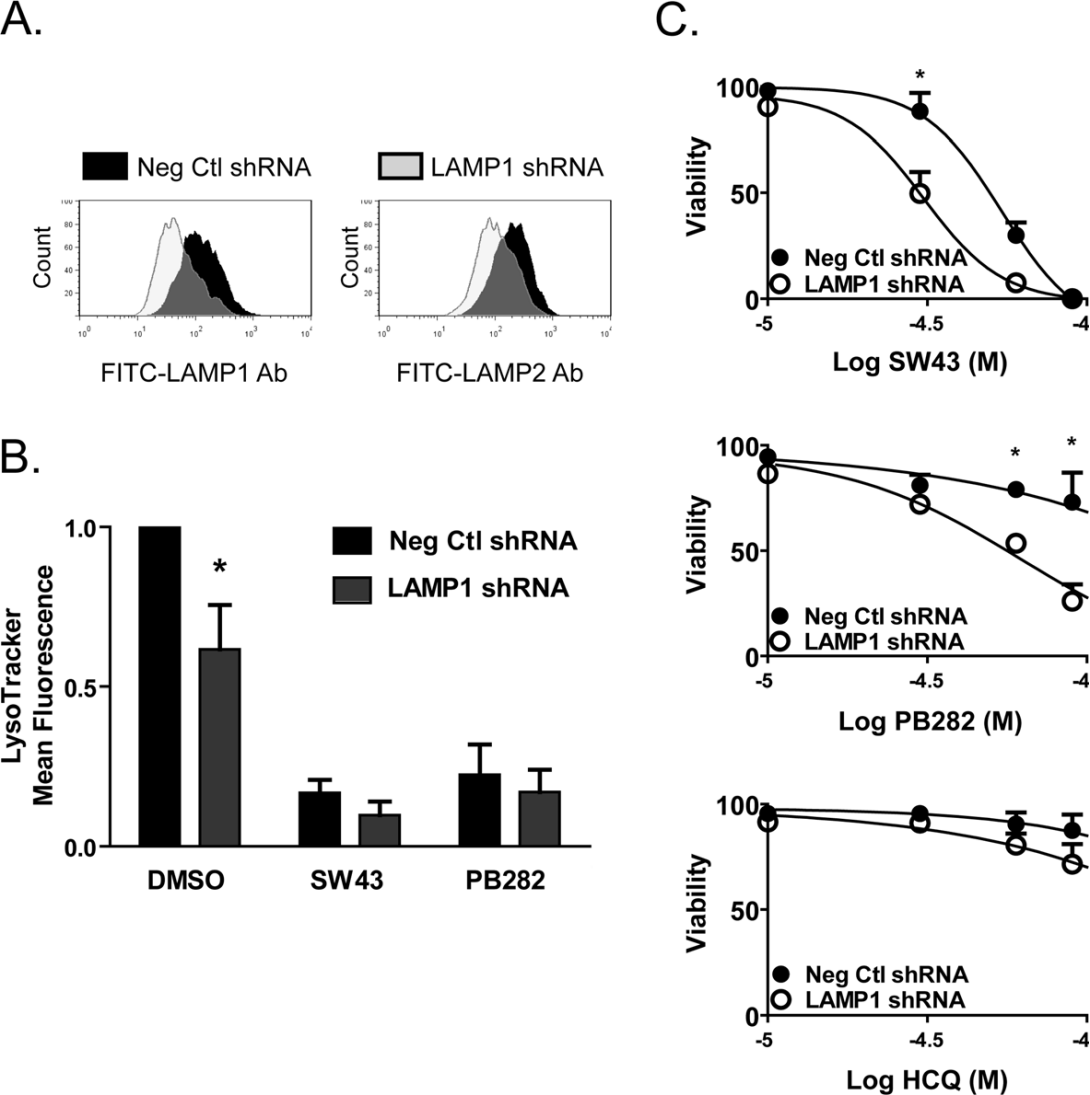
** Sensitization to lysosomal membrane permeabilization and cell deathby LAMP1 shRNA.** (**A**) Bxpc3 cells transformed with pLKO.1-LAMP1 or pLKO.1-Neg Ctl confirmed for knockdown of LAMP1/2 by flow cytometry. (**B**) Lysosomal retention of LysoTracker Green by flow cytometry of transformed cells treated with DMSO vehicle, SW43, or PB282 for one hour. Data represents mean fluorescence normalized to DMSO treated pLKO.1-Neg cells, n = 3. (**C**) Viability of transformed cells following 24 hour treatment with SW43, PB282, HCQ. Data represents percent viability compared to DMSO treated cells, n = 3, * p < 0.05.

LAMP1 and LAMP2 are large, closely homologous, glycoprotein constituents of the lysosomal membrane that contribute to protection of the membrane against the acidic enviroment within this organelle [21]. We hypothesized that decreasing the content of LAMP1 in the lysosome would subject the membrane to increased stress and susceptibility to permeabilization. pLKO.1-LAMP1 and pLKO.1-Neg shRNA lentiviral constructs were used to transform and select Bxpc3 (Figure 4A) cells with decreased expression of LAMP1 and LAMP2 (Figure 4A). LAMP1 shRNA-expressing cells significantly retained less fluorescence of LysoTracker Green (Figure 4B), mean fluorescence 61.6 ± 0.1 percent of vehicle, with moderate decreases following treatment with SW43 or PB282. LAMP1 knockdown significantly increased susceptibility of Bxpc3 cells to cell death following treatment with SW43 and PB28, with less protection observed in the lower range of toxicity with HCQ (Figure 4C).

### Lysosomal accumulation of sigma-2 receptor ligands is required for LMP and cell death

**Figure 5:**
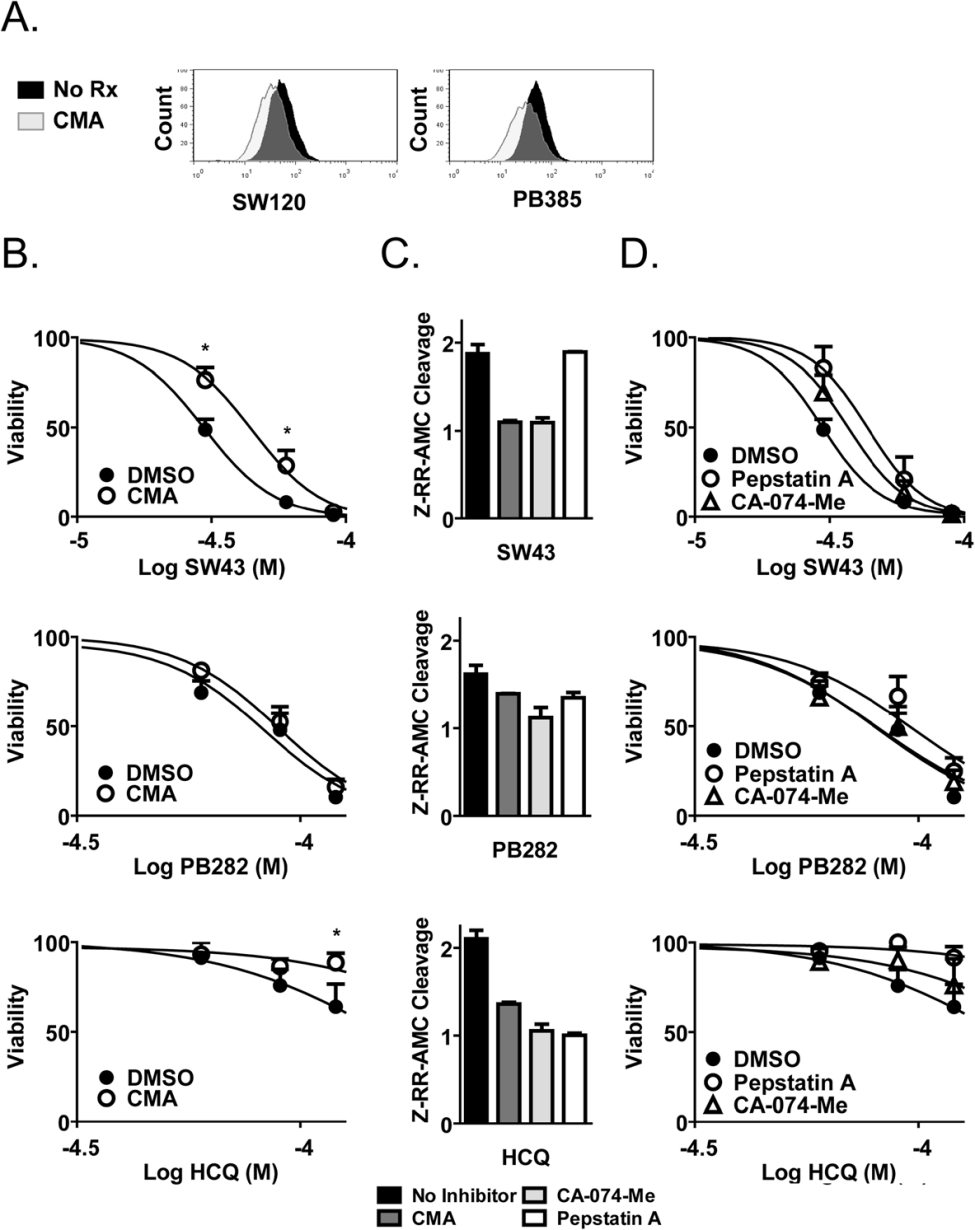
** Sigma-2 receptor ligand-mediated cell death is dependent on lysosomal accumulation and membrane permeabilization.** (**A**) Accumulation of sigma-2 receptor ligands SW120 and PB385 in Bxpc3 cells following inhibition of lysosomal pH gradient with the V-ATPase inhibitor concanamycin A (CMA) (10 nM) detected by flow cytometry. (**B**) Cell viability following 24 treatment with sigma-2 receptor ligands SW43 and PB282 or lysosomal detergent hydroxychlorquine (HCQ) in the presence of CMA (10 nM). Data represents percent viability compared to DMSO treated cells, n = 3, p < 0.05 (**C**) Cleavage of fluorescent peptidase substrate Z-RR-AMC following one hour treatment with SW43 (30 μM), PB282 (30 μM), or HCQ (60 μM), in the presence of CMA (10 nM) or peptidase inhibitors CA-074-Me (10 μM) and pepstatin A (100 μM). Data is relative to DMSO treated cells and is representative of experiments performed in triplicate. (**D**) Cell viability following 24 hour treatment with SW43, PB282, or HCQ in the presence of CA-074-Me or pepstatin A. Data represents percent viability compared to DMSO treated cells, n = 3.

Bxpc3 cells were treated with CMA (10 nM) for 60 minutes in order to effectively inhibit the pH gradient across the lysosomal membrane. Subsequent accumulation of the fluorescently labeled sigma-2 receptor ligands SW120 and PB385 showed marked decrease of fluorescence intensity by flow cytometry (Figure 5A). Bxpc3 cells pretreated with CMA were more viable following treatment with sigma-2 ligands, but the response was greater for SW43 and HCQ compared to PB282 (Figure 5B). To determine whether LMP lead to release of proteases into the cytoplasm, the cytosolic fraction was isolated from the lysosomal fraction by selective permeabilization of the plasma membrane with digitonin, and cleavage ofcathepsin B substrate Z-RR-AMC was assessed. All compounds increased Z-RR-AMC cleavage within one hour of treatment, and CMA decreased this Z-RR-AMC cleavage to baseline (Figure 5C). CA-074-Me and pepstatin A decreased cleavage for all compounds as well, with the exception that pepstatin A was not observed to inhibit cleavage following SW43 treatment. Functional rescue of viability in the presence of CA-074-Me and pepstatin A was assessed at 24 hours following treament, and pepstatin A was observed to rescue viability across a titrated dose range for all compounds, while CA-074-Me had a lesser effect, though the observed differences did not reach statistical significance (Figure 5D).

### Antioxidants are protective of cellular toxicity

**Figure 6:**
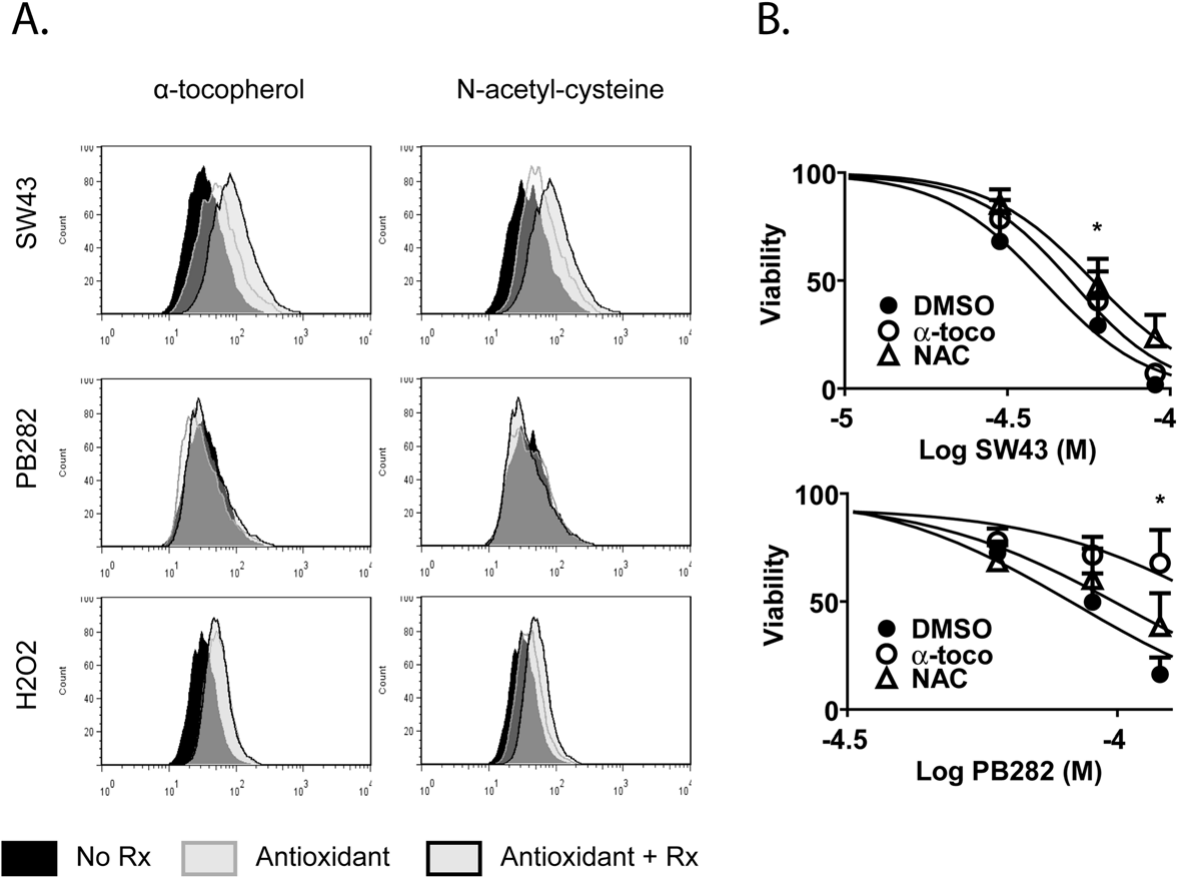
** Antioxidants are protective of cellular toxicity.** (**A**) ROS detection by flow cytometry in Bxpc3 cells with 5-(and-6)-chloromethyl-2',7'-dichlorodihydrofluorescein diacetate, acetyl ester (CM-H2DCFDA) following 24 hour treatment with SW43 (60 μM), PB282 (90 μM), or hydrogen peroxide (H2O2, 100 μM) in the presence of lipophilic antioxidant α-tocopherol (α-toco) or hydrophilic antioxidant N-acetylcyteine (NAC). (**B**) Cell viability following 24 hour treatment with SW43 or PB282 in the presence of α-toco or NAC. Data represents percent viability compared to DMSO treated cells, n = 3, * p < 0.05.

Sufficient LMP leads to apoptosis through release of specific mediators of crosstalk with the mitochondria such as cathepsins, and release or stimulation of reactive oxygen species (ROS) [22]. Though cancer cells typically have a higher than normal content of ROS due to relative anoxia, additional oxidative stress is lethal due to oxidation and disruption of membrane lipids, proteins, and DNA [23]. To assess the involvement of ROS in apoptosis following sigma-2 receptor ligand treatement, we examined the influence of antioxidants on cell death. ROS production in Bxpc3 cells following 24 hour treatment with SW43 (60 μM), PB282 (90 μM), and H_2_O_2_(100 μM) was detected with 5-(and-6)-chloromethyl-2',7'-dichlorodihydrofluorescein diacetate acetyl ester (CM-H_2_DCFDA) as described in the Materials and Methods. Substantial amounts of ROS were detected with SW43 and H_2_O_2_, but no ROS was detectable after treatment with PB282. ROS was decreased following SW43 treatment in the presence of antioxidants α-tocopherol (α-toco) and n-acetylcysteine (NAC), while ROS from H_2_O_2_ was only decreased by NAC (Figure 6A). The impact of antioxidants on cell viability was assessed following 24 hour treatment with SW43 and PB282. Antioxidants protected against sigma-2 receptor ligand induced cell death, with NAC protecting against SW43 to a greater extent than α-toco. Interestingly, while PB282 treatment did not result in detectable ROS release, both antioxidants increased tumor cell viability after PB282 exposure (Figure 6B).

### Caspase-3 inhibition by lipophilic antioxidant correlates with caspase dependence

**Figure 7:**
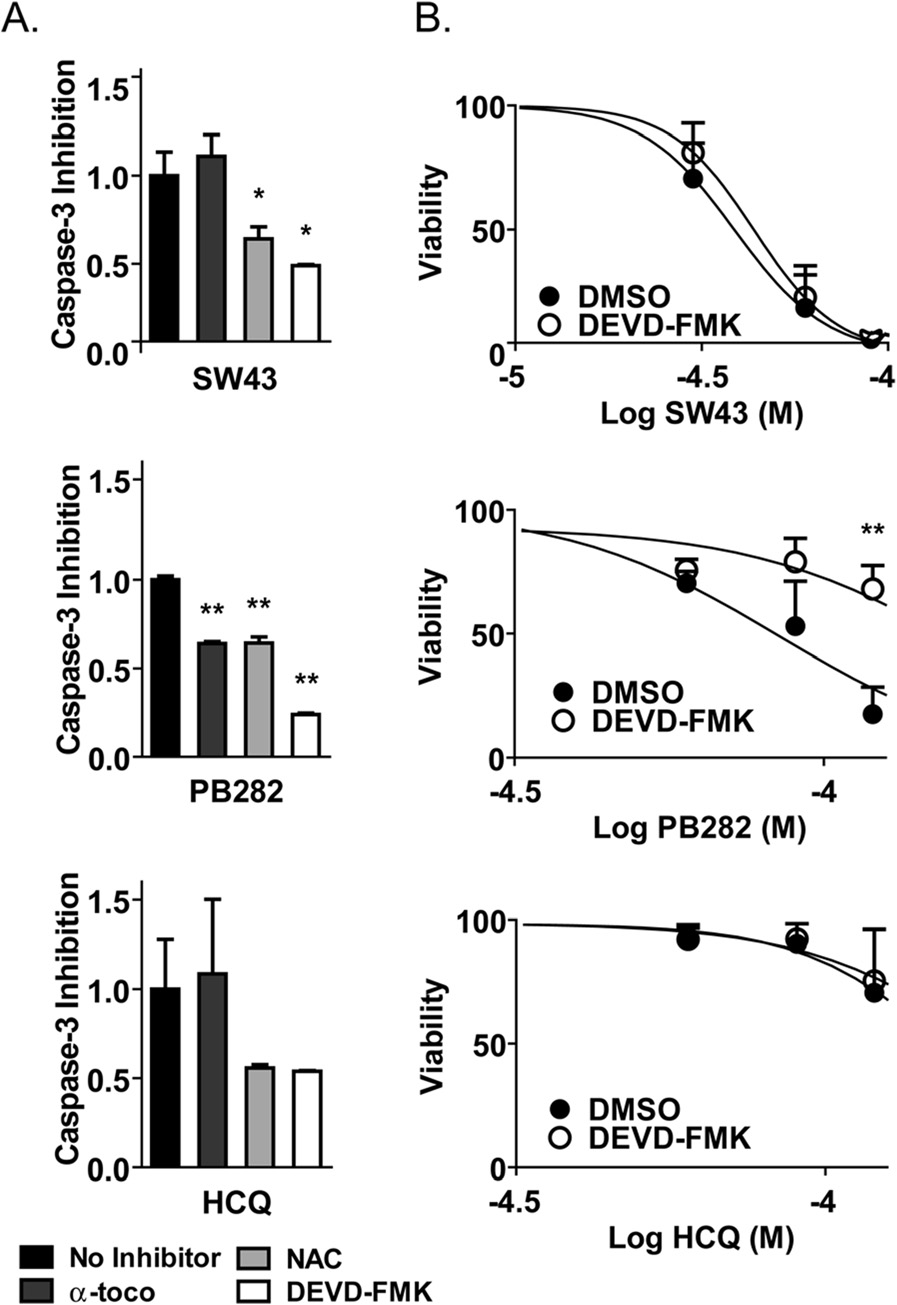
** Caspase-3 inhibition by lipophilic antioxidant correlates with caspase dependence.** (**A**) Caspase-3 inhibition by the hydrophobic antioxidant α-tocopherol (α-toco), hydrophilic antioxidant N-acetylcyteine (NAC), or caspase-3 inhibitor DEVD-FMK (1 μM) in Bxpc3 cells following 24 hour treatment with SW43 (30 μM), PB282 (90 μM), or HCQ (90 μM). Data represents normalized inhibition compared to caspase-3 inducing treatment, n = 3, p < 0.05. (**B**) Cell viability following 24 hour treatment with SW43 or PB282 in the presence of α-toco or NAC. Data represents percent viability compared to DMSO treated cells, n = 3, * p < 0.05.

Caspase-3 has been extensively studied as a mechanism of sigma-2 receptor ligand mediated apoptosis, and we wished to examine the impact of ROS stimulation by structurally different ligands. Basal caspase-3 activity by SW43, PB282, and HCQ treatment following 24 hours was detected by cleavage of Z-DEVD-AMC as previously described [10] (Figure 7A). This activation was inhibited by α-toco following PB282 treatment, but not following SW43 or HCQ treatment. NAC, however, decreased caspase-3 activation by all compounds. DEVD-FMK caspase-3 inhibitor was used as a positive control for inhibition in all experiments. One hour pretreatment with DEVD-FMK, followed by 24 hour treatment with SW43, PB282, or HCQ showed little protection for SW43 and HCQ, while PB282 mediated cytotoxicity was protected to a much greater extent following caspase-3 inhibition (Figure 7B). Interestingly, caspase-3 activity was not observed in Aspc1 cells (Additional file 3 figure S3C), a cell line with less sensitivity to PB282 (Additional file 3 figure S3D).

## Discussion

Recent synthesis of fluorescently labeled analogs of SV119 (SW120) and PB28 (PB385), allowing live cell imaging, has shown sigma-2 receptor ligand subcellular localization to the membrane components of the cell ultrastructure [16,17]. In various pancreatic cancer cell lines we have observed similar results, and hypothesized that strong uptake into the endo-lysosomal compartment induces lysosomal membrane permeabilization (LMP). In addition, weakly basic amines as a class of drugs have been shown to induce LMP [24] and cell death [25], and the amine groups present on sigma-2 receptor ligands suggest they can induce LMP. We examined here whether this could influence the caspase-3 activation in pancreatic cancer we observed earlier [8-10] and found that LMP occurs shortly following treatment with a variety of structurally diverse sigma-2 receptor ligands, verified by both AO and LysoTracker release from the lysosome.

Uptake of fluorescently labeled compounds was inhibited by blocking the lysosomal pH gradient with concanamycin A (CMA), a specific inhibitor of the V-Type ATPase [26,27], and translated into significant viability protection following treatment. SW43 was a stronger inducer of LMP, with greater protection from CMA pretreatment than for PB282. This that some sigma-2 receptor ligands have a greater propensity to influence the lysosomal death pathway Chemical structure differences may be responsible for this difference. For instance, the structure of the *N*-(9-(6-Aminohexyl)-9-azabicyclo[3.3.1]-nonan-3α-yl)-*N*-(2-methoxy-5-methylphenyl) carbamate hydrochloride (SV119) derivatives contain an alkyl extension with terminal amine group that is not present in the 1-cyclohexyl-4-[3-(5-methoxy-1,2,3,4-tetrahydro-naphthalen-1-yl)-propyl]-piperazine dihydrochloride (PB28) derivatives, a moiety that increases lysosomal membrane insertion and permeabilization [28].

The lysosomal associated membrane proteins 1 and 2 (LAMP1/2) are homologous proteins of the lysosomal membrane [21,29,30], where they are highly glycosylated and to contribute to protection of the lysosomal membrane and its proteins from the hostile constituents such as hydrogen ion and proteases [31]. In addition, down-regulation of LAMP1/2 have been previously shown to sensitize cells to lysosomal mediated death pathways [32], and we wished to confirm that sigma-2 receptor ligands act through a component of this pathway by decreasing LAMP1 expression with a lentivirus driven shRNA in Bxpc3 cells. Transformed cells had weaker lysosomes that retained less LysoTracker and the effect was additive with sigma-2 receptor ligand. Overall LysoTracker Green uptake was decreased as assessed by flow cytometry, which could have occurred by either a decreased number of lysosomes, or increased leakage across the membrane. We believe that the enhanced killing of transformed cells was due to compromise of the membrane integrity rather than decreased number of lysosomes based on the above finding that sigma-2 ligand accumulation in lysosomes is a necessary component of cell death.

LMP mediated cell death has been extensively studied recently in the context of apoptosis induction in cancer cells [22,33,34]. The exact mechanism of LMP is still undetermined, and whether it involves pore formation or selective movement of contents, dyes of increasing molecular weight and size can be differentially released indicating some selectivity to LMP. A large number of known inducers of LMP exist, reviewed in [22], and culminate in the release of proteases such as cathepsin B, D, and L, amonst others. Following treatment with sigma-2 receptor ligands, or hydroxychloroquine, we observed a near doubling of Z-RR-AMC cleavage within one hour, which was inhibited completely by CMA and CA-074-Me, supporting the above finding that uptake of the compound into the lysosome is a critical step in LMP mediated cell death.

Cancer cells can undergo both caspase-dependent and independent pathways of cell death following LMP, depending on the degree of insult [22]. Cathepsins mediate crosstalk between the lysosome to the mitochondria [35], where a caspase-dependent pathway is stimulated with cytochrome c release and superoxide production [36]. With larger insults, a caspase-independent death pathway may be followed with release of cathepsins, cytosolic acidification, and caspase-2 activation [22]. ROS production due to either pathway can act as both an effector and initiator of cell death. Amongst known inducers of LMP, oxidative stress itself ultimately leads to lipid peroxidation of the membrane with permeabilization [37]. Thus, production of ROS following treatment can amplify LMP.

Protection against ROS can be by antioxidants or intracellular enzymes such as superoxide dimutase, catalase, and glutathione peroxidase. NAC is an small diffusible, hydrophobic antioxidant that is a precursor to glutathione, a cellular thiol-reducing agent oxidized by glutathione peroxidase in the reduction of hydrogen peroxide to water. In this study, NAC protected against cell death by SW43 to a greater extent than α-toco, while α-toco protected against PB282 more than NAC. While the mechanism of α-toco protection against oxidative stress is thought to be by prevention of membrane lipid peroxidation, and NAC as a general reducing agent, we believe this indicates key differences in the intracellular sites exposed to oxidative stress by sigma-2 receptor ligands. Intracellular ROS was detected with CM-H_2_DCFDA following SW43, but not PB282. This was decreased by both α-toco and NAC following SW43 treatment, but only with NAC following H_2_O_2_, suggesting that H_2_O_2_treatment did not induce oxidative stress in the membranes where the α-toco is present, while SW43 may have. PB282 viability protection by antioxidants is through a mechanism other than inhibiting oxidative stress.

Alpha-tocopherol has been previously established to protect cells from sigma-2 mediated mitochondrial ROS production and caspase-3 release [10,38,39], and in this study we observed that caspase-3 stimulated by PB282 was inhibited in the presence of this antioxidant, while it did not protect that from SW43 or HCQ. In addition, caspase-3 inhibitor DEVD-FMK provided ample protection against cell death following PB282 treatment, but little following SW43 or HCQ despite detectable caspase-3 activity. The observation that the Aspc1 cell line did not induce caspase-3 activity following sigma-2 receptor ligand treatement, but retained cytotoxicity following lysosomal membrane permeabilization following SW43 treatment, further suggests the susceptibility differences are through slighty convergent pathways. Thus, it is most likely that PB282 undergoes caspase-dependent cell death following LMP that is mediated through a mitochondrial pathway, protected by α-toco. Conversely, SW43 undergoes caspase-independent cell death following LMP, with oxidative stress playing a stronger role in cell death.

## Conclusions

Structurally diverse compounds with high affinity to sigma-2 receptors are effective in decreasing tumor burden in preclincial models of human pancreatic cancer. While caspase-3 has been shown to be activated following treatment with this class of compounds, conflicting reports exist on caspase-3 dependence or independence for cytotoxicity. We suggest that caspase-3 dependence may be influenced by lysosomal mediated oxidative stress in a compound specific manner amongst sigma-2 receptor ligands. Better understanding of the susceptibility of cancers to certain death pathways will ultimately allow tailoring of sigma-2 receptor ligand treatment choice.

## Materials and Methods

### Cell Culture

Cell lines were maintained in RPMI media (GIBCO) supplemented with L-glutamine (2 mM), (HEPES) (1 mM), pyruvate (1 mM), sodium bicarbonate (0.075 % w/v), penicillin and streptomycin (100 IU/mL), amphotericin (0.25 μg/mL), and 10 % fetal bovine serum (Atlanta Biologicals, Lawrenceville, GA). Cells were seeded at a density of 2 x 10^5^/mL unless otherwise stated and maintained in a humidified atmosphere of 5 % CO_2_ at 37°C.

### Compounds

Sigma-2 receptor ligands were synthesized as previously described [10,16,17,40-42]. The imaging dyes acridine orange and LysoTracker Red were obtained from Invitrogen (Carlsbad, CA), FITC mouse anti-human CD107a (LAMP1) and CD107b (LAMP2) antibodies from BD Biosciences (Franklin Lakes, NJ), peptidase inhibitors CA-074-Me and pepstatin A, and fluorogenic peptidase substrate Z-RR-AMC from Enzo Life Sciences (Plymouth Meeting, PA), caspase-3 inhibitor Z-DEVD-FMK from R&D Systems (Minneapolis, MN); caspase-3 substrate Ac-DEVD-AMC from Bachem Biosciences, Inc (King of Prussia, PA); All other reagents were obtained from Sigma-Alrich (St. Louis, MO) unless otherwise stated. Compounds were dissolved in DMSO with final concentrations less than 0.3 %.

### In vivo tumor treatment

Athymic nude mice from Harlan Bioproducts, Inc. were inoculated subcutaneously with 1x10^6^ Bxpc3 cells in the right flank. Tumor sizes were monitored with calipers and when tumors reached an average of 5 mm in diameter, mice were randomized and treated daily with equimolar doses of sigma-2 receptor ligands SV119 (1.0 mg), SW43 (1.1 mg), PB28 (0.9 mg), or PB282 (0.9 mg) resuspended in vehicle consisting of 5 % DMSO, 5 % EtOH, and 10 % Cremophor in 1X PBS and injected intraperitoneally. Data represents mean ± SEM, n = 7–10 per group.

### Confocal microscopy

Cells grown on glass cover slips were incubated with SW120 or PB385 (100 nM) in the presence of LysoTracker Red (25 nM) for 30 minutes at 37°C. Cells were washed with PBS and fixed in 2 % paraformaldehyde for 30 minutes at 37°C prior to additional washing and mounting with ProLong Gold antifade reagent. Confocal imaging was performed on a Carl Zeiss Axiovert 100 inverted microscope, fitted with LSM 510 laser scanning microscope camera and software. Images were collected with filter bandwidths corresponding to 505–530 nm for green, 560–615 nm for red, and > 650 nm for far red, with 4 scans over 11.8 seconds.

### Fluorescence microscopy

Cells grown on glass cover slips were loaded with acridine orange (2 μg/mL) for 15 minutes at 37°C prior to treatment for one hour with compounds. Cover slips were inverted onto slides and images taken immediately at 40X magnification on anOlympus BX51 microscope fitted with a U-LH100HE reflective fluorescence system and equipped with a Diagnositic Instruments, Inc. SPOT camera and software. Chroma Technology Corp filter sets were used for green (exciter: D480/30x, emitter: 535/40 m, beamsplitter: 505dclp), red (exciter: D540/25x, emitter: 606/55 m, beamsplitter: 556dclp), and blue (exciter: D360/40x, emitter: 460/50 m, beamsplitter: 400dclp). Scale bar equals 20 μm.

### Dye retention analysis by flow cytometry

Cells were incubated with acridine orange (2 μg/mL) or LysoTracker Green (25 nM) for 30 minutes at 37°C prior to treatment with compounds for one hour. Cells were washed and mean fluorescence quantified with a FACSCalibur flow cytometer (BD Biosciences, San Jose, CA). Mean fluorescence was normalized to DMSO to determine the degree of lysosomal permeabilization. In addition, cells were pretreated with concanamycin A (10 μM) for one hour at 37°C prior to staining with either SW120 or PB385 (100 nM) for 30 minutes at 37°C and the difference in uptake represented by histogram.

### Constructs

shRNAlentiviral constructs in pLKO.1 against human LAMP1 was purchased from Sigma Aldrich, and following verification of knockdown, clone ID NM_005561.2-1183s1c1 used to compromise lysosomal integrity. Packaging vectors were obtained through Addgene, Inc. (Cambridge, MA). Lentivirus particles were prepared by transfection of 293 T cells in T75 flasks with 3 μg construct, 2.8 μgpRSV-Rev, 2.4 μgpMDLg/pRRE, and 0.6 μg pMD2.G utilizing FuGENE® 6 Transfection Reagent from F. Hoffmann-La Roche Ltd. (Basel, Switzerland). Forty-eight and 72 hours following transfection, supernatant was transferred to Bxpc3 cells in the presence of polybrene (8 μg/mL). Transformed cells were selected with puromycin (1 μg/mL) and assayed accordingly.

### Antibody staining

Cells were washed once with PBS prior to fixation with IC Fixation Buffer (eBiosciences) for 15 minutes at 37°C. Fixed cells were washed with PBS, resuspended in Permeabilization Buffer (eBiosciences), and incubated for 30 minutes at room temperature. Intracellular antigen staining was performed with FITC-antibody dilution of 1:100 in Permeabilization Buffer for 60 minutes at room temperature. Mean fluorescence in FL1 was quantified with a FACSCalibur flow cytometer.

### Cell viability

Cell lines maintained at optimal culture conditions were seeded into 96-well white, clear-bottom plates and following treatment, viability determined with CellTiter-Glo Luminescent Viability Assay from Promega (Madison, WI). Luminescence was quantified with a SpectraMax Gemini microplate spectrofluorometer from Molecular Devices (Silicon Valley, CA). Viability relative to vehicle was fit by non-linear regression and plotted against concentration.

### Cellular protease assay

Cells were treated in the presence of inhibitors and cytosolic extracts prepared using the digitonin extraction method as previously described [43]. Washed cells were resuspended at 1x10^6^ cells/mL in extraction buffer consisting of sucrose (250 mM), HEPES (20 mM), KCl (10 mM), MgCl_2_ (1.5 mM), EDTA (1 mM), and digitonin (30 μM). Cells were placed on ice on an orbital shaker for 10 minutes prior to centrifugation for 1 min at 14,000 rpm at 4°C. Supernatants were collected and 20 μL used to detect cleavage of Z-RR-AMC in and equal volume of reaction buffer consisting of sodium acetate (100 mM), NaCl (200 mM), EDTA (4 mM), DTT (10 mM), and Z-RR-AMC (10 μM). Plates were read following incubation at 37 ° for 60 minutes with SpectraMax Gemini microplate spectrofluorometer, Molecular Devices (Silicon Valley, CA) (ex 355 nm, em 450 nm).

### Detection of reactive oxygen species (ROS) by flow cytometry

Cells were seeded into 12-well plates one day prior to treatment, stained with 25 μM 5-(and-6)-carboxy-2’,7’-dichlorodihydro-fluorescein diacetate (carboxy-H_2_DCFDA) (Image-iT Live Green Reactive Oxygen Species Detection Kit, Molecular Probes, Eugene, OR) for 30 minutes at 37°C and treated overnight. Fluorescence intensity (max 529 nM) was quantified in the FL1 channel with a FACSCalibur flow cytometer.

### Caspase-3 activity

Cells were maintained at optimal conditions and seeded in 96-well black-bottom plates in a volume of 100 μL. Following treatment, 5X assay buffer containing EDTA (10 mM), CHAPS (5 %), HEPES (100 mM), DTT (25 mM), and Ac-DEVD-AMC (250 μM) was added directly to the cell media and incubated for two hours at 37°C on a microplate shaker, and liberated AMC quantified with a SpectraMax Gemini microplate spectrofluorometer, Molecular Devices (ex 355 nm, em 450 nm). Caspase-3 activity is normalized to the absence of inhibitor.

### Statistical analysis

Statistical analysis and data plotting was conducted using GraphPad Prism (GraphPad Software, San Diego, CA). Data represents the mean ± SEM. Viability IC_50_ values at 18 hours were calculated by line fitting normalized viability versus concentration with non-linear regression and statistical significance determined using one-way ANOVA. Differences in viability, caspase-3 activity, apoptosis, and oxidation status were analyzed using two-way ANOVA to identify differences and confirmed with paired two-tailed t-tests. Blood cytology and biochemistry results were analyzed using one-way ANOVA with Tukey’s multiple comparison test. Statistical analysis for the difference in tumor volume between treatments groups was determined with the repeated measures ANOVA. Kaplan-Meier survival curves were plotted and differences compared with a log-rank test. A p-value of less than 0.05 was considered significant for all tests.

## Abbreviations

(LMP), Lysosomal membrane permeabilization; (NAC), N-acetylcysteine; (α-toco), α-tocopherol; (AO), acridine orange; (CMA), concanamycin A; (HCQ), hydroxychloroquine; (DMSO), dimethyl sulfoxide; (ROS), reactive oxygen species; (PB385), 6-[1-tetrahydronaphthalen-1-yloxy]-N-(7-nitrobenzo-2-oxa-1,3-diazol-4-yl)hexanamine; (SW120), N-9-(10-(7-nitrobenzo-2-oxa-1,3-diazol-4-ylamino)decyl)-9-azabicyclo[3.3.1]nonan-3a-yl-N-(2-methoxy-5-methylphenyl) carbamate; (SV119), N-(9-(6-aminohexyl)-9-azabicyclo[3.3.1]nonan-3α-yl)-N-(2-methoxy-5-methylphenyl) carbamate hydrochloride; (SW43), N-(9-(10-aminohexyl)-9-azabicyclo[3.3.1]nonan-3α-yl)-N-(2-methoxy-5-methylphenyl) carbamate hydrochloride; (PB28), 1-cyclohexyl-4-[3-(5-methoxy-1,2,3,4-tetrahydro-naphthalen-1-yl)propyl]-piperazine dihydrochloride.

## Competing interests

No authors of this manuscript have any competing interests to disclose.

## Authors’ contributions

JRH participated in the design and conduction of experiments, data analysis, and final drafting and writing of the manuscript. SV, RHM, CA, and FB all contributed new reagents for these experiments. PG and DS were involved in research design and contributed to the drafting of the manuscript. WGH was closely involved in research design and drafting of the final manuscript. All authors read and approved the final manuscript

## Supplementary Material

Additional file 1 **Figure S1.** In vivo efficacy of sigma-2 receptor ligands. Female C57BL/6 mice inoculated subcutaneously with 1x106 Panco2 cells were treated daily with sigma-2 receptor ligands when tumors reached an average of 5 mm in diameter. Data represents mean ± SEM, n = 7–10 per group. Mice received daily treatment through the duration presented. Click here for file

Additional file 2 **Figure S2.** Colocalization of SW120 and PB385 in Bxpc3 and Aspc1 pancreatic cancer cell lines by fluorescence microscopy. Live cells were imaged following incubated with LysoTracker Red (50 nM), red, and fluorescent sigma-2 receptor ligand (500 μM), green, for 30 minutes at 37°C prior to nucleic acid counterstaining with Hoechst, blue, scale bar = 20 μm. Click here for file

Additional file 3 **Figure S3.** Lysosomal membrane permeabilization comparison between Bxpc3 and Aspc1 pancreatic cancer cell lines. (A) Acridine orange (2 μg/mL) staining for lysosomal integrity by fluorescence microscopy in Bxpc3 cells, top row, and Aspc1, bottom row, treated with vehicle, PB282 (30 μM), SW43 (30 μM), or CMA (10 nM) for one hour, scale bar = 20 μm. Flow cytometric analysis of acridine orange stained cells following treatment with sigma-2 receptor ligands, CMA, or HCQ as positive control. FL3 = orange, FL1 = green. (B) Confirmation of lysosomal membrane permeabilization with LysoTracker Green following same treatments as above in Bxpc3 and Aspc1 cells. (C) Overall caspase-3 activity compared between Bxpc3 and Aspc1 cell lines following treatment with SW43 (30 μM), PB282 (90 μM), or HCQ (90 μM). (D) Viability of Aspc1 cells following 24 hour treatment with SW43, PB282, or HCQ. Data represents percent viability compared to DMSO treated cells, n = 3, * p < 0.05. Click here for file
