# Supplementary figures and images for: Lysosomal Membrane Permeabilization is an Early Event in Sigma-2 Receptor Ligand Mediated Cell Death in Pancreatic Cancer
Source: J Exp Clin Cancer Res. 2012 May 2;31(1):41. doi: 10.1186/1756-9966-31-41 (PMC3414770; doi:10.1186/1756-9966-31-41)

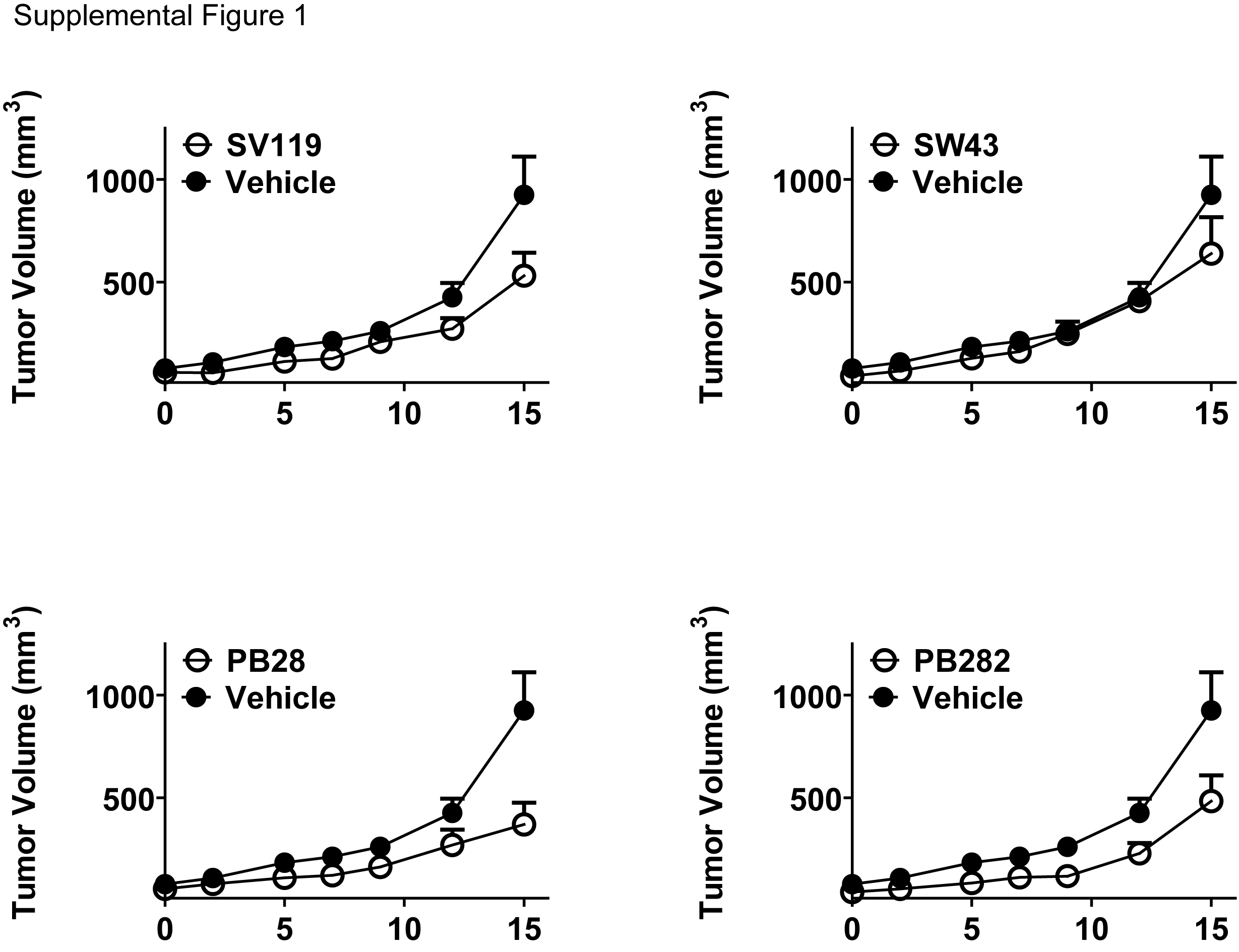

Supplement: Additional file 1 — Figure S1. In vivo efficacy of sigma-2 receptor ligands. Female C57BL/6 mice inoculated subcutaneously with 1x10^6^ Panco2 cells were treated daily with sigma-2 receptor ligands when tumors reached an average of 5 mm in diameter. Data represents mean ± SEM, n = 7–10 per group. Mice received daily treatment through the duration presented. [file 1756-9966-31-41-S1.tiff]

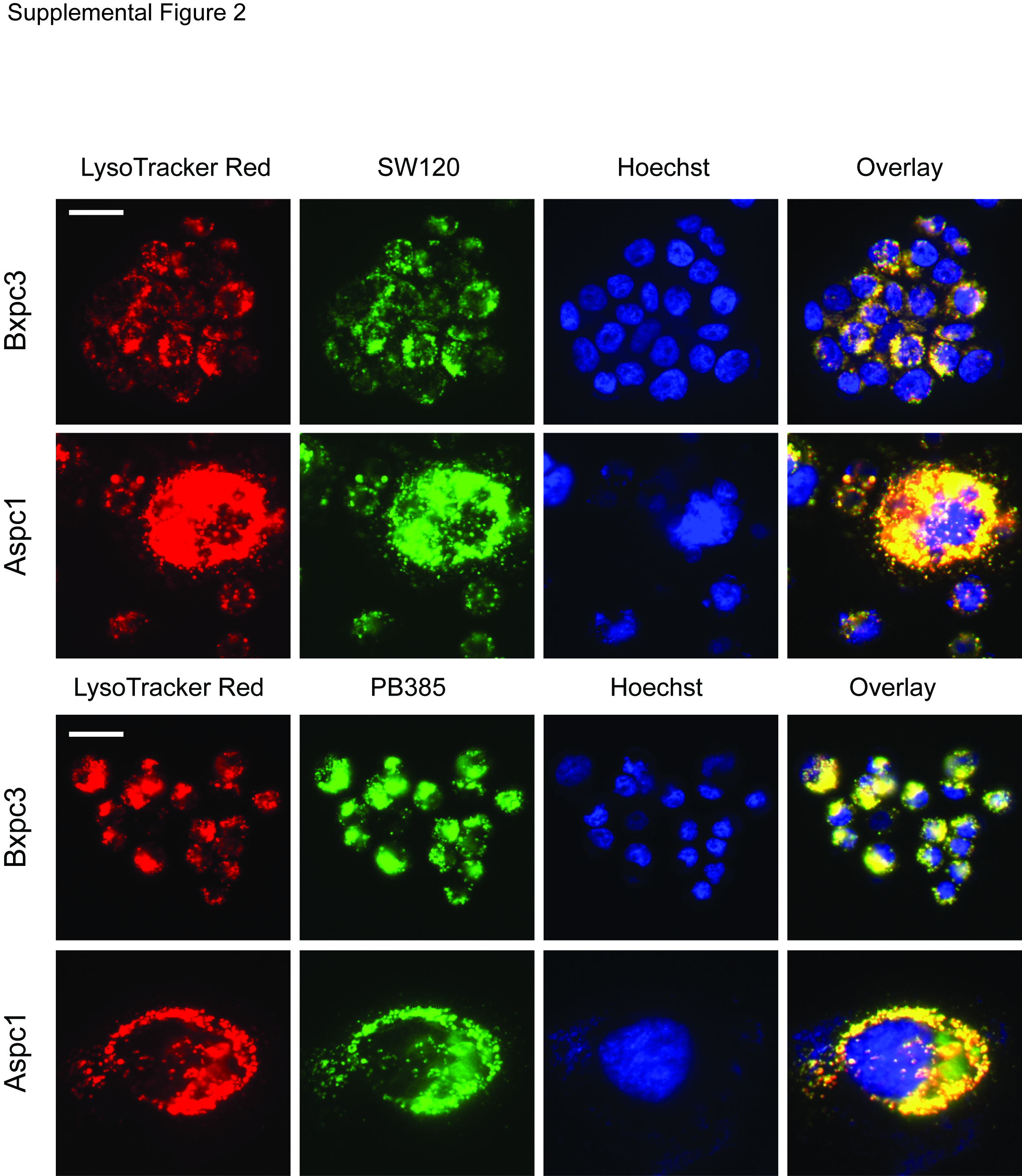

Supplement: Additional file 2 — Figure S2. Colocalization of SW120 and PB385 in Bxpc3 and Aspc1 pancreatic cancer cell lines by fluorescence microscopy. Live cells were imaged following incubated with LysoTracker Red (50 nM), red, and fluorescent sigma-2 receptor ligand (500 μM), green, for 30 minutes at 37°C prior to nucleic acid counterstaining with Hoechst, blue, scale bar = 20 μm. [file 1756-9966-31-41-S2.jpeg]

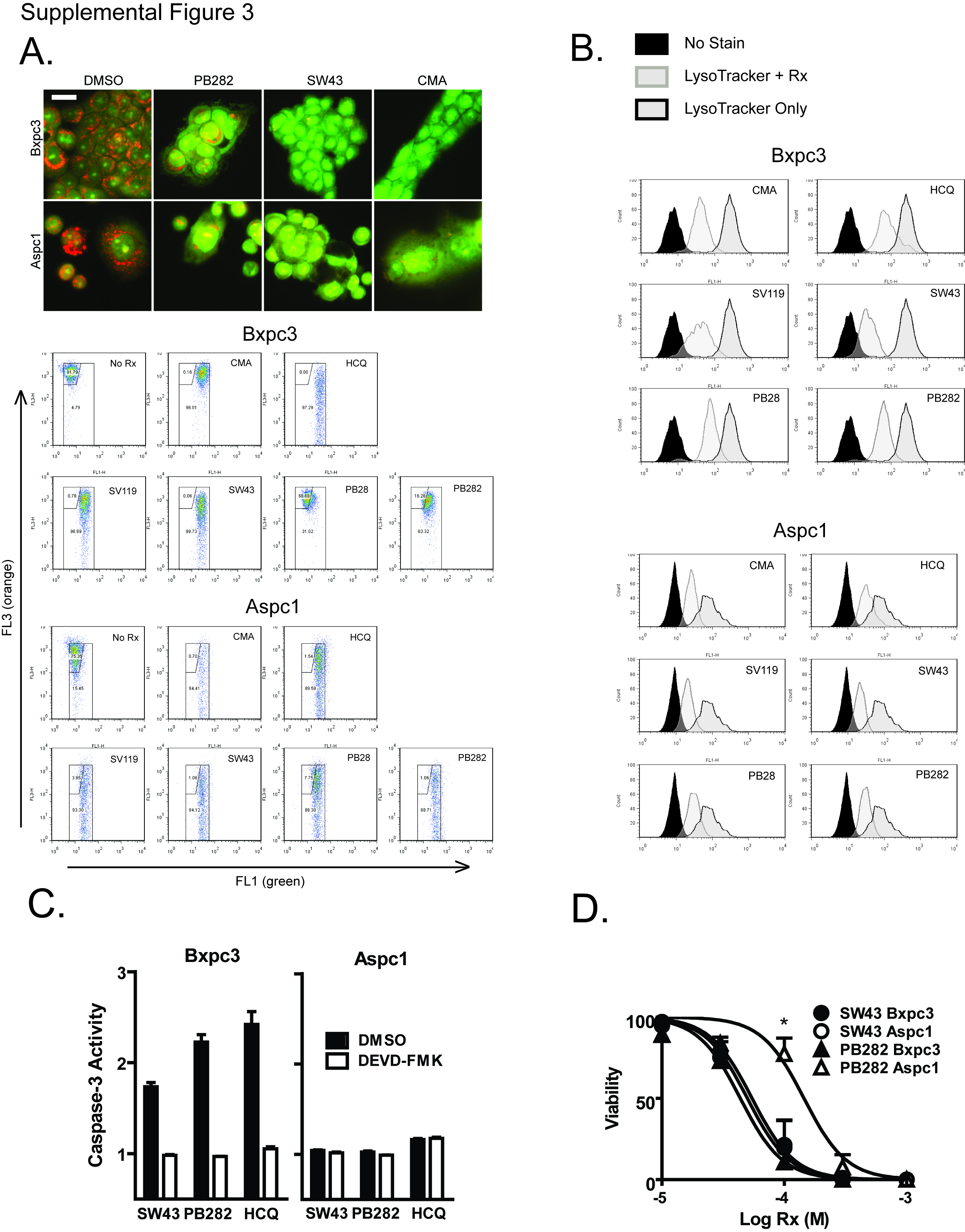

Supplement: Additional file 3 — Figure S3. Lysosomal membrane permeabilization comparison between Bxpc3 and Aspc1 pancreatic cancer cell lines. (A) Acridine orange (2 μg/mL) staining for lysosomal integrity by fluorescence microscopy in Bxpc3 cells, top row, and Aspc1, bottom row, treated with vehicle, PB282 (30 μM), SW43 (30 μM), or CMA (10 nM) for one hour, scale bar = 20 μm. Flow cytometric analysis of acridine orange stained cells following treatment with sigma-2 receptor ligands, CMA, or HCQ as positive control. FL3 = orange, FL1 = green. (B) Confirmation of lysosomal membrane permeabilization with LysoTracker Green following same treatments as above in Bxpc3 and Aspc1 cells. (C) Overall caspase-3 activity compared between Bxpc3 and Aspc1 cell lines following treatment with SW43 (30 μM), PB282 (90 μM), or HCQ (90 μM). (D) Viability of Aspc1 cells following 24 hour treatment with SW43, PB282, or HCQ. Data represents percent viability compared to DMSO treated cells, n = 3, * p < 0.05. [file 1756-9966-31-41-S3.jpeg]
